# 지역사회 거주 노인의 COVID-19 관련 염려와 우울이 손 씻기 수행도에 미치는 영향: 2020년 지역사회건강조사

**DOI:** 10.7586/jkbns.24.003

**Published:** 2024-02-27

**Authors:** Suyoung Choi, Jung Jae Lee, Moonju Lee, Jeong Yun Park, Yong Taek Yoon, Hyo Jeong Song

**Affiliations:** 1 College of Nursing, Health and Nursing Research Institute, Jeju National University, Jeju, Korea; 1 제주대학교 간호대학, 제주대학교 건강과간호연구소; 2 School of Nursing, University of Hong Kong, Hong Kong; 2 School of Nursing, University of Hong Kong; 3 School of Nursing, UT Health San Antonio; 3 School of Nursing, UT Health San Antonio, TX, USA; 4 Department of Clinical Nursing, University of Ulsan, Ulsan, Korea; 4 울산대학교 임상전문간호학; 5 Department of Philosophy, College of Humanities, Jeju National University, Jeju, Korea; 5 제주대학교 인문대학 철학과

**Keywords:** Aged, Depression, Infection control, Hand hygiene, 노인, 우울, 감염관리, 손 씻기

## Abstract

**Purpose:**

This study investigated hand-washing practice among community-dwelling older adults during the coronavirus disease 2019 (COVID-19) pandemic and aimed to identify the impact of COVID-19-related concerns and depression on hand-washing practice.

**Methods:**

This was a secondary analysis of data extracted from the 2020 Community Health Survey. The primary data were collected through self-reporting from August 10 to September 8, 2020 in a cross-sectional study. The subjects comprised of 1,350 adults aged 65 or older living in Jeju Province who participated in the 2020 Community Health Survey.

**Results:**

The factors affecting hand-washing practice among older adults were male older adults (β = –.18, *p* < .001), age (β = –.07, *p* = .001), no education (β = –.20, *p* < .001) and elementary, middle, and high school graduation (β = –.15, *p* < .001) compared to a college or higher education, poor health perception (β = –.13, *p* < .001), COVID-19-related concerns (β = .08, *p* = .005), and depression (β = –.07, *p* = .001). To summarize, the factors negatively affecting hand-washing practice included male gender, lower education level, poor health perception, and depression. In contrast, factors positively associated with hand-washing practice included COVID-19-related concerns.

**Conclusion:**

These findings show the importance of considering these multifaceted determinants when designing targeted interventions and educational programs to promote hand-washing among older adults. Additionally, based on the relationship between hand-washing practice and COVID-19-related concerns and depression, interventions that can alleviate mental problems along with providing proper education are required.

## 서론

### 1. 연구의 필요성

한국의 65세 이상 노인인구는 2022년도에 전체 인구의 17.4%에서 2050년에는 40.1%까지 증가할 것으로 예측되고 있다[1]. 전 세계적으로 급속히 증가하는 노인인구에 대한 건강정책의 방향은 기능상태가 악화하여 거동이 불편하더라도 생활환경에서 적절한 주거 및 사회적 지원이 제공된다면 노인이 거주하던 곳에서 독립적으로 생활할 수 있도록 지지하는 ‘지역사회 계속 거주’의 방향으로 추진되고 있어, 지역사회 내에서 노인이 자존감을 유지하면서 삶의 질과 안녕을 누리면서 살아가는 것에 대한 초점이 강조되고 있다[2]. 우리나라의 지역사회 내 노인복지관, 경로당 등의 시설은 노인들이 쉽게 방문하여 친구들을 만나 친목을 나누고 식사, 취미활동 등을 공유하는 장소일 뿐만 아니라, 취약한 노인을 대상으로 상담 및 정보제공, 기능회복, 급식, 식사배달 등의 건강지원 및 건강관리를 받을 수 있는 장소로도 기능하고 있다[3]. 그러나 2020년 SARS-Cov-2감염으로 인한 coronavirus disease 2019 (이하 COVID-19)의 확산으로 지역사회 노인복지관, 경로당 등의 운영이 중단되었고, 취약한 노인들의 사회적 활동 및 건강관리의 기회가 제한되었다[3]. 이로 인해 COVID-19 감염의 유행은 노인인구에서 신체적 건강 문제와 함께 불안, 우울 등 정신적 건강 문제를 악화시킬 수 있다는 우려가 제기되었다[4]. 세계보건기구(World Health Organization, WHO)는 지역사회 내에서의 사회활동 참여는 개인의 신체적, 정신적 기능과 관련이 있다고 하였고[5], 선행연구에서도 사회활동은 노인의 정신 기능 및 신체적 활동을 좋게 하는 긍정적 효과를 가진다고 보고하였다[6].

2019년 12월부터 COVID-19의 범 유행은 빠르게 진행되어 전 세계로 확산되었고, 특히 노년층에서의 발병 위험과 높은 사망률로 인해 건강 전략으로서 바이러스의 확산을 피하기 위해 검역 및 사회적 격리를 하게 되었다[7]. 사회적 격리는 특히 노인의 정신적 건강에 부정적 영향을 미쳤고, 실제로 COVID-19 기간 동안의 우울증 유병률이 14.6% [8]~47.2% [9]로 다양하게 보고되었다. 노인에 있어서 우울은 삶의 의욕을 떨어뜨리고 살고자 하는 의지를 무력화시킬 수 있으며, 일상생활에서 자가간호 및 사회적 활동을 하지 않음으로써 정신적, 신체적 건강 상태에 손상을 초래할 수 있다[10].

WHO는 전 세계의 COVID-19 감염 실태를 보고하였고, 2020년 7월 31일 기준으로 216개 국가 및 지역에서 총 17,064,064건의 확인 사례와 668,073명의 사망자가 발생되었다[11]. COVID-19의 감염 증상은 경미한 독감 유사 증상부터 심각한 폐렴에 이르기까지 다양한 증상으로 호흡기 감염을 일으키며, 당뇨병, 심장 질환, 폐 또는 신장 질환을 앓는 환자에게서 이환 및 중증 상태로 진행할 위험이 높았다[2,12]. 특히 노인의 경우 만성질환의 동반과 함께 약화된 면역체계로 인해 COVID-19 감염에 취약하다[13]. 각 국가들은 감염의 확산을 줄이기 위한 방법으로서 올바른 손 씻기를 생활화하도록 권장하였으나[14], 실제로 사회적 거리두기로 인해 각 의료기관 및 보건소 시설들과 차단된 상황에서 지역사회 거주 노인들이 올바른 손 씻기의 중요성을 이해하고, 손 씻기의 방법을 정확하게 익히고 수행했는지는 잘 알려지지 않았다.

COVID-19 팬데믹 기간 동안 특히 노년층에서 감염률과 사망률이 높았고, 의료시설 폐쇄와 이용 제한, 대중교통의 제한 등의 사회적 격리 상황에서 노인들은 감염에 대한 두려움과 함께 불안, 우울을 경험하였다[15]. 불안과 우울은 자가관리 및 COVID-19 감염을 예방하기 위한 손 씻기 등의 예방 행위 수행에 영향을 미칠 수 있는데, 노인을 대상으로 시행한 연구에서 COVID-19 감염에 대한 위험을 지각하는 수준이 높은 경우 손 씻기, 사회적 거리두기와 같은 감염 예방 행위 수행률이 높았다[16,17]. 반면 우울이 동반된 경우 고혈압과 당뇨병 환자[18], 일반 성인[19]과 노인[17]을 대상으로 시행한 연구에서 손 씻기 수행률이 낮은 것으로 보고되었다.

손 씻기는 세균을 제거하고, 질병 예방 및 세균이 다른 사람에게 퍼지는 것을 방지하는 가장 효과적인 방법 중 하나이며[20], 손 씻기를 통해 피부접촉, 호흡기 또는 음식을 통해 전염되는 바이러스나 박테리아 감염의 위험을 줄이고 완화하는 것으로 알려져 있다[21,22]. 따라서 본 연구에서는 COVID-19 팬데믹 시기에 이루어진 2020년 지역사회건강조사 자료를 바탕으로 지역사회 거주 노인이 경험한 COVID-19 관련 염려와 우울 정도가 손 씻기 수행도에 미치는 영향을 파악하고자 한다. 손 씻기 수행은 향후 새로운 병원체 및 변종 바이러스 감염이 발생하는 경우에 중요한 방어선 역할을 하게 되며, 감염에 대한 노인의 건강을 보호하기 위해 지속적으로 유지되어야 한다[23]. 본 연구에서는 제주 지역의 거주하는 노인들의 특성을 고려하여 추후 특화된 손 씻기 교육 프로그램을 위한 기초자료로 제공하고자 본 연구를 시도하였다.

### 2. 연구의 목적

1) 노인의 일반적 특성에 따른 손 씻기 수행도를 파악한다.

2) 노인의 COVID-19 관련 염려와 우울 정도가 손 씻기 수행도에 미치는 영향을 파악한다.

## 연구 방법

### 1. 연구 설계

본 연구는 COVID-19 유행 기간 동안 지역사회 거주 노인의 손 씻기 수행도를 파악하고, COVID-19 관련 염려와 우울 정도가 손 씻기 수행도에 미치는 영향을 파악하기 위하여 2020년 지역사회건강조사의 제주 지역의 원시자료를 활용한 단면조사연구이다.

### 2. 연구 대상

본 연구 대상은 2020년 지역사회건강조사의 제주 지역 참여자 5,055명에서 65세 이상의 노인 1,525명을 선정하였고, 이중 손 씻기 수행에 응답을 한 1,350명으로 정하였다[23].

### 3. 연구 도구

#### 1) 일반적 특성

일반적 특성은 성별, 연령, 교육수준, 배우자 유무, 직업 유무, 체질량지수, 지각된 건강상태로 구성되었다. 교육수준은 ‘무학,’ ‘초등·중등·고등학교,’ ‘대학교이상’의 3개 군으로 구분하였고, 직업은 ‘최근 1주일 동안 수입을 목적으로 1시간 이상 일을 하거나, 18시간 이상 무급 가족 종사자로 일한 적이 있는가’ 문항에 ‘예’로 응답한 경우 직업이 있는 것으로 보았다. 지각된 건강상태는 매우 좋음과 좋음을 합하여 ‘좋음,’ ‘보통,’ 그리고 매우 나쁨과 나쁨을 합하여 ‘나쁨’으로 구분하였다.

#### 2) COVID-19 관련 염려

2020년 질병관리청 지역사회건강조사에서 조사된 ‘COVID-19 관련 염려’는 총 5문항으로 구성되었다[24]. 문항별 내용은 ‘COVID-19에 감염될까 염려된다,’ ‘코로나19에 감염되면 죽을 수 있을까 염려된다,’ ‘COVID-19에 감염되면 그 이유로 주변으로부터 비난이나 피해를 받을 것 같은 염려된다,’ ‘가족 중 건강 취약자가 감염될까 염려된다,’ ‘COVID-19 유행으로 인해 나와 가족의 경제적 피해가 올까 염려된다’로 구성되었다. 각 문항별 점수는 ‘전혀 그렇지 않다’ 1점에서 ‘매우 그렇다‘ 5점으로, 총 점수는 5점에서 25점의 범위를 가지며, 점수가 높을수록 COVID-19 관련 염려 정도가 심함을 의미한다. 본 연구에서 도구의 신뢰도는 Cronbach α .81로 나타났다.

#### 3) 우울

우울은 1999년에 Spitzer 등[25]이 개발한 Patient Health Questionnaire (PHQ-9)로 Choi 등[26]이 국문으로 번역한 자기보고식 설문도구이다. 본 도구는 최근 2주 동안 개인이 우울이나 절망감, 피로 등을 경험한 정도를 나타내는 총 9문항으로 구성되었고, 문항별 점수는 ‘전혀 아니다,’ 0점에서 ‘거의 매일,’ 3점으로 이루어졌다. 총 점수 범위는 0-27점으로서 점수가 높을수록 우울 정도가 심각하다는 것을 의미한다. Choi 등[26]의 도구 신뢰도는 Cronbach’s α .85였고, 본 연구의 도구 신뢰도는 Cronbach α .77로 나타났다.

#### 4) 손 씻기 수행도

2020년 질병관리청 지역사회건강조사[24]에서 사용된 손 씻기 수행 도구는 총 6문항으로 구성되었다. 본 연구에서는 손 씻기 수행을 ‘최근 1주일 동안 식사하기 전,’ ‘화장실 다녀온 후,’ ‘외출에서 돌아와서,’ ‘평소 손을 씻을 때 30초 이상 흐르는 물에 꼼꼼히 씻는지,’ 얼마나 자주 하는지를 묻는 4문항과 ‘평소 손을 씻을 때 비누나 손세정제를 얼마나 자주 사용하는지’에 대한 1문항을 포함하여 총 5문항을 사용하였다. 이 중 4개 문항의 문항별 점수는 4점 척도로 ‘거의 씻지 않았다,’ 1점에서 ‘항상 씻었다,’ 4점으로 구성되었고, 1개 문항은 ‘비누나 손세정제를 전혀 사용하지 않는다’ 1점에서 ‘항상 사용한다’ 5점 척도이지만, 본 연구에서는 ‘전혀 사용하지 않는다’와 거의 사용하지 않는다‘를 합해 1점으로 산출하여 4점 척도로 수정하였다. 본 도구의 총 점수는 최소 5점에서 최대 20점의 범위를 가지며, 점수가 높을수록 손 씻기 수행도가 높은 것을 의미한다. 본 연구의 도구 신뢰도는 Cronbach α .83으로 나타났다.

### 4. 자료 수집

본 연구는 2020년 지역사회건강조사를 활용한 이차 분석조사이다. 지역사회건강조사는 지역보건법에 의거하여 질병관리청과 광역자치단체 및 255개 시·군·구 보건소, 34개 대학교가 지역사회건강조사를 전담하는 지속가능한 수행체계를 구축하여 수행하는 국가승인통계 조사이다. 2020년 지역사회건강조사는 COVID-19 감염이 유행하는 2020년 8월 16일부터 10월 31일까지 이루어졌다. 조사대상은 조사시점에 표본가구에 거주하는 만 19세 이상의 성인(2001년 7월 31일 이전 출생자)이며, 조사단위는 가구(가구조사) 및 가구원(개인조사)이다. 지역사회건강조사는 조사원이 표본가구를 방문하여 노트북에 탑재된 전자조사표를 이용해 조사대상자와 1:1 면접 조사(computer assisted personal interviewing) 방식으로 수행되었다. 본 연구는 제주 지역의 65세 이상 지역사회 거주 노인에서 손 씻기 수행에 응답한 자료를 사용하였다.

### 5. 자료 분석

본 자료는 SAS (version 9.2 for Windows) 프로그램을 사용하여 분석하였다. 일반적 특성, COVID-19 관련 염려, 우울, 손 씻기 수행도는 빈도, 평균과 표준편차, 정규성 검정은 서술적 통계를 사용하였다. 일반적 특성에 따른 손 씻기 수행도는 independent t-test, Analysis of Variance (ANOVA)와 ANOVA 후 집단 간의 차이는 Duncan test를 실시하였고, 손 씻기 수행도와 COVID-19 관련 염려, 우울 간의 상관관계는 Pearson’s correlation coefficients로 산출하였다.

손 씻기 수행도에 영향을 미치는 요인을 파악하기 위해 stepwise multiple regression을 실시하였으며, 분석에 투입된 변수는 일반적 특성에 따른 손 씻기 수행도에 유의한 차이를 나타낸 성별, 연령, 교육수준, 지각된 건강상태와 손 씻기 수행도와 유의한 상관관계를 나타낸 COVID-19 관련 염려와 우울이었고, 성별, 교육수준, 지각된 건강상태는 가변수로 처리하였다. 통계적 유의수준은 *p* < .05로 하였다.

### 6. 윤리적 고려

본 연구는 제주대학교 생명윤리심의위원회의 승인(IRB No. JJNU-IRB-2022-013)을 받았으며, 질병관리청 지역사회건강조사 부서로부터 원시자료를 받아 이 연구를 수행하였다.

## 연구 결과

### 1. 일반적 특성

본 연구의 남성은 628명(46.5%), 여성은 722명(53.5%)이었다. 평균 연령은 73.98 ± 6.51세로 65-74세가 772명(57.2%), 75-84세 478명(35.4%), 85세 이상 100명(7.4%)의 순으로 나타났다. 배우자와 함께 거주하는 경우는 941명(69.7%)이었고, 직업은 730명(54.1%)이 가지고 있었다. 교육수준은 무학이 172명(12.7%), 초등·중·고등학교 졸업 1,060명(78.6%), 대학 졸업 이상은 117명(8.7%)이었고, 지각된 건강상태는 ‘나쁨’ 352명(26.1%), ‘보통’ 529명(39.2%), ‘좋음’ 469명(34.7%)로 나타났다. 체질량지수는 25 kg/m^2^ 이상인 경우가 445명(33.0%)이었다(Table 1).

### 2. 일반적 특성에 따른 손 씻기 수행도 차이

일반적 특성에 따른 손 씻기 수행도는 성별(t = –3.76, *p* = .001), 연령(F = 1.22, *p* = .017), 교육수준(F = 12.24, *p* < .001), 지각된 건강상태(F = 16.27, *p* < .001)에 따라 통계적으로 유의한 차이가 있었다. 성별에 따른 손 씻기 수행도는 여성이 남성보다 손 씻기 수행도가 높았고, 연령은 65-74세 군과 75-84세 군보다 85세 이상군에서 유의하게 손 씻기 수행도가 낮았다. 교육수준에 따른 손 씻기 수행도는 무학이 가장 낮았고, 초등·중·고등학교 졸업, 대학 졸업 이상의 순으로 높게 나타났다. 지각된 건강상태에 따른 손 씻기 수행도는 ‘좋음’과 ‘보통‘에 비해 ’나쁨‘에서 유의하게 낮았다(Table 2).

### 3. 손 씻기와 COVID-19 관련 염려, 우울 간의 상관관계

손 씻기 수행도는 평균 16.99 ± 2.96점(범위 2∼25점), COVID-19 관련 염려는 19.09 ± 4.18점(범위 5∼20점), 우울은 1.87 ± 2.63점(범위 0∼21점)이었다(Table 3).

손 씻기 수행도와 COVID-19 관련 염려, 우울 간의 상관관계를 보면, COVID-19 관련 염려 정도가 높을수록 손 씻기 수행도가 높았고(r = .10, *p* = .037), 우울 정도가 높을수록 손 씻기 수행도가 낮았다(r = –.11, *p* < .001) (Table 4).

### 4. 손 씻기 수행도에 미치는 영향요인

손 씻기 수행도에 영향을 미치는 요인을 파악하기 위해 독립변수에 대한 회귀분석의 가정을 검정하였고, 다중공선성을 확인한 결과 허용도(tolerance)는 0.39∼0.97로 0.1이상이고, 분산팽창인자(variance inflation factor)가 1.03∼2.58로 기준이 되는 10을 넘지 않아 다중공선성의 문제는 없는 것으로 확인되었다.

손 씻기 수행도에 영향을 미치는 주요 요인은 남성(β = –.18, *p* < .001), 연령(β = –.07, *p* = .001), 무학(β = –.20, *p* < .001)과 초등·중·고등학교 졸업(β = –.15, *p* < .001), 지각된 건강상태가 나쁨(β = –.13, *p* < .001), COVID-19 관련 염려(β = .08, *p* = .005), 우울(β = –.07, *p* = .001)로 나타났다. 즉 남성이 여성보다 손 씻기 수행도가 낮았고, 연령이 많을수록 손 씻기 수행도가 낮았다. 교육수준이 대학교 졸업 이상인 군에 비해 무학과 초등·중·고등학교 졸업 군이 손 씻기 수행도가 낮았고, 지각된 건강상태가 ‘나쁨’군이 ‘좋음‘군에 비해 손 씻기 수행도가 낮았다. COVID-19 관련 염려가 높을수록 손 씻기 수행도가 높았고, 우울이 높을수록 손 씻기 수행도가 낮았다. 손 씻기 수행도에 대한 이들 변인의 영향력은 총 7%로 설명하였다(F = 13.55, *p* < .001) (Table 5).

## 논의

본 연구는 COVID-19 팬데믹 시기에 이루어진 2020년 지역사회건강조사 자료를 바탕으로 제주 지역사회 거주 노인의 COVID-19 관련 염려, 우울 정도가 손 씻기 수행도에 미치는 영향을 파악하고자 시도되었다.

본 연구에서 손 씻기 수행도에 영향을 미치는 요인은 성별, 연령, 교육수준, 지각된 건강상태, COVID-19 관련 염려, 우울로 나타났다. 성별로 보면 남성 노인은 여성에 비해 손 씻기 수행도가 낮은 것으로 나타났고, 이것은 국외 5개국 성인을 대상으로 한 설문조사에서 여성이 남성에 비해 손 씻기를 더 잘하는 것으로 나타난 Anderson-Carpenter와 Tacy [14]의 연구결과와 일치하였다. 또한 COVID-19 팬데믹 기간 중 성인 및 노인을 포함하여 수행한 조사에서도 손 씻기에 대한 지식, 태도와 수행에서 남성이 여성에 비해 낮은 수준을 나타났다[27]. 따라서 남성에서 손 씻기 수행도가 여성보다 낮은 요인들을 파악하고, 이를 기본으로 여성과 남성 노인에게 적합한 손 씻기 교육중재가 이루어져야 할 것이다.

본 연구에서 노인의 연령이 많을수록 손 씻기 수행도가 낮은 것으로 나타났는데, 반면 Lu 등[16]의 연구에서는 대상자의 연령이 많을수록 COVID-19 감염에 대한 높은 위험을 인지하고 이것은 감염 예방을 위한 손 씻기, 마스크 착용, 사회적 거리두기와 같은 예방적 수행을 높게 한다고 하였다. 일반적으로 높은 연령으로 갈수록 노인은 이동의 제한, 인지 저하, 관절염 또는 시각 장애로 인해 손 씻기를 위해 움직이거나, 올바른 손 씻기 수행을 하는데 어려움을 가질 수 있고, 따라서 이러한 특성으로 인해 본 연구에서 수행도가 낮게 나타난 것으로 생각한다. 노인은 연령이 많아질수록 신체 기능의 저하, 만성건강문제, 면역 약화 등으로 감염에 대한 취약성이 높아질 수 있기 때문에 효과적인 손 씻기를 생활화 하는 것이 더욱 중요하다.

본 연구에서 노인의 교육수준은 손 씻기 수행도와 관련이 있었고, 대학교 졸업이상에 비해 무학과 초등·중·고등학교 졸업자의 손 씻기 수행도가 낮은 것으로 나타났다. 선행 연구에서도 교육수준이 낮을수록 손 씻기 수행도가 낮았다고 보고하였다[27]. 노인의 교육수준은 COVID-19 팬데믹 상황에서 감염예방과 관리에 대한 지식을 습득하거나 이해하는데 영향을 미칠 수 있으며[14], 노인의 교육수준이 높을수록 감염예방에 대한 손 씻기 수행의 중요성을 잘 알고 인식을 함으로써 자연히 수행도가 높았을 것으로 생각한다[28]. Smail 등[29]의 연구에서 노인들이 가정에서 손 씻기, 마스크 착용 등 예방적 방역수칙에 대한 미디어 방송을 많이 청취할수록 COVID-19 예방을 위한 지식을 얻게 되었고, 이는 손 씻기를 높게 수행하였던 것과 관련이 있었다고 하였다. 따라서 추후 신종 감염병 상황이 발생하거나, 혹은 일상생활에서 감염성 질환의 예방을 위해 손 씻기 교육과 중재를 하는 경우 각 노인의 교육수준을 고려하는 것은 매우 중요하다고 본다.

본 연구에서 노인이 지각한 건강상태는 손 씻기 수행도와 관련이 있었다. 지각된 건강상태가 나쁜 군에서 손 씻기 수행도가 낮았고, 건강상태를 좋다고 지각하는 군에서 손 씻기를 높게 하는 것으로 나타났다. 자신의 건강상태를 좋다고 지각하는 것은 손 씻기와 같은 감염예방 행위를 긍정적으로 수행하도록 원동력이 되었다고 생각된다. 본 연구와 다른 결과를 나타낸 선행 연구에서는 노인이 자신의 건강상태를 좋지 않다고 지각하는 경우 스스로 감염에 대한 높은 위험을 느끼고, COVID-19 감염에 대항하는 손 씻기 행위를 더 잘 하는 것으로 보고하였다[16]. 한편 본 연구에서 노인이 COVID-19 관련 염려를 높게 할수록 손 씻기 수행도가 높게 하였고, 이는 노인이 COVID-19와 같은 감염병에 이환되는 것에 대한 염려와 위험을 느끼는 것 또한 감염 예방 활동을 수행할 수 있는 긍정적인 자극으로 작용할 수 있음을 보여주고 있다. 그러나 염려와는 다르게 본 연구에서 노인의 우울 정도가 심각할수록 손 씻기 수행도가 낮게 나타났고, 선행 연구에서도 우울은 노인의 손 씻기와 예방적 수행 활동에 부정적으로 작용하고 있었다[16,18]. COVID-19 팬데믹 기간 동안 사회적 거리 두기는 노인에게 앉아 있는 시간의 증가와 신체 활동 감소, 그리고 정신적으로 불안과 우울 등 정신건강 문제를 경험하도록 하였다[30]. 이러한 고조된 우울은 노인의 동기 부여, 손 씻기를 포함한 자기 관리에 부정적인 영향을 미쳤고, 따라서 우울을 잘 조절하는 것은 손 씻기 수행의 준수나 전반적인 자기관리를 높일 수 있다[27]. 노인의 정신건강 지지는 회복력을 촉진하고 COVID-19를 포함한 감염에 맞서 싸우는 신체의 능력을 향상시킬 수 있다. 따라서 COVID-19 팬데믹과 같은 상황이 재현될 경우에 대비하여 지역사회 보건소 및 의료기관을 중심으로 노인의 사회적 고립을 줄이고 우울과 같은 정신건강 문제를 예방할 수 있는 포괄적인 중재 마련이 요구된다. 즉, 규칙적인 신체활동, 적절한 수면과 건강한 식사 관리, 인지훈련, 유연성 운동과 같은 건강교육 프로그램과 함께 정신건강 지원을 위한 상담서비스, 지지그룹 프로그램 등을 포함하되 물리적 거리두기가 필요한 상황에서도 서비스가 지속될 수 있도록 온라인 플랫폼, 원격서비스 등을 통해 올바른 손 씻기 방법 등의 건강관리 교육이 진행될 수 있어야 한다.

본 연구의 손 씻기 수행도에 미치는 요인들의 영향력은 7%로 낮게 나타났고, 이는 2020년 지역사회건강조사의 자료를 이차분석을 하는데 있어서 노인들의 특성을 고려한 변수 선정이 충분하지 않았다고 생각된다. 추후 본 결과를 기반으로 노인의 손 씻기 수행도에 미치는 관련 특성들을 파악하기 위해 체계적 문헌고찰이 요구된다고 본다.

본 연구에서는 제주 지역에 거주하는 노인들의 특성을 고려하여 추후 손 씻기 수행을 높이기 위한 교육을 중재하는데 자료로 제공하고자 의도하였으나, 한편으로는 본 연구 결과를 일반화하는데 제한점이 있다. 또한 본 연구는 단면조사 연구이기 때문에 손 씻기 수행도에 대한 인과관계를 파악할 수 없다는 연구의 제한점을 가진다.

## 결론

본 연구에서 지역사회 거주 노인의 손 씻기 수행도에 영향을 미치는 요인은 성별, 연령, 교육수준, 지각된 건강상태, COVID-19 관련 염려, 우울로 나타났다. 즉, 남성의 경우와 연령이 많을수록 손 씻기 수행도가 낮았고, 교육수준이 낮을수록, 그리고 지각된 건강상태를 나쁘게 지각할수록 손 씻기 수행도가 낮았다. 노인의 COVID-19 관련 염려가 높을수록 손 씻기 수행도가 높았고, 우울이 높을수록 손 씻기 수행도가 낮았다. 손 씻기는 감염에 대항하여 중요한 방어선 역할을 하고, 노인의 건강을 보호하기 위해서는 올바른 손 씻기 수행은 지속적으로 유지되어야 한다. 노인의 손 씻기 수행을 높이기 위하여 교육수준이 낮거나 고연령, 성별의 차이 등을 고려하여 맞춤화된 교육중재가 이루어져야 한다. 또한 노인의 우울과 같은 정신건강 문제를 완화하고 잘 관리함으로써 손 씻기의 수행도를 높이는 데 기여할 수 있다.

## Figures and Tables

**Table 1.** General Characteristics of Subjects (N = 1,350)

Variables	Categories	n (%)	M ± SD
Sex	Men	628 (46.5)	
	Women	722 (53.5)	
Age (yr)	65-74	772 (57.2)	73.98 ± 6.51
	75-84	478 (35.4)	
	≥ 85	100 (7.4)	
Living with spouse	Yes	941 (69.7)	
	No	409 (30.3)	
Employment	Yes	730 (54.1)	
	No	620 (45.9)	
Education	None	172 (12.7)	
	Elementary·middle or	1,060 (78.6)	
	high school		
	≥ College	117 ( 8.7)	
Perceived health status	Poor	352 (26.1)	
	Moderate	529 (39.2)	
	Good	469 (34.7)	
Body mass index (kg/m^2^)	< 18.5	54 (4.0)	23.81 ± 3.11
	18.5-24.9	851 (63.0)	
	≥ 25	445 (33.0)	

M = mean; SD = standard deviation.

**Table 2.** Hand-washing Practice by General Characteristics (N = 1,350)

Variables	Categories	Hand washing		
		M ± SD	t or F	*p* ^†^
Sex	Men	16.67 ± 3.05	–3.76	.001
	Women	17.27 ± 2.85		
Age (yr)	65-74	17.26 ± 2.74^a^	1.22	.017 (a < b)
	75-84	16.87 ± 3.01^a^		
	≥ 85	15.45 ± 3.77^b^		
Living with spouse	Yes	17.05 ± 2.87	1.06	.290
	No	16.86 ± 3.16		
Employment	Yes	16.91 ± 3.03	–1.05	.294
	No	17.08 ± 3.24		
Education	None	16.22 ± 3.13^a^	12.24	< .001 (a < b < c)
	Elementary·middle or	17.01 ± 2.97^b^		
	high school			
	≥ College	17.95 ± 2.32^c^		
Perceived health status	Poor	16.26 ± 3.19^a^	16.27	< .001 (a < b)
	Moderate	17.11 ± 2.84^b^		
	Good	17.41 ± 2.82^b^		
Body mass index	≤ 18.5	16.80 ± 2.95	1.11	.621
(kg/m^2^)	18.5-24.9	16.93 ± 3.06		
	≥ 25	17.13 ± 2.77		

M = mean; SD = standard deviation.

† Duncan test.

**Table 3.** Levels of COVID-19-related Concerns, Depression, and Hand-washing Practice (N = 1,350)

Variables	M ± SD	Range	Minimum	Maximum
COVID-19-related concerns	19.09 ± 4.18	5-20	5	20
Depression	1.87 ± 2.63	0-27	0	21
Hand-washing practice	16.99 ± 2.96	5-20	2	25

COVID-19 = coronavirus disease 2019; M = mean; SD = standard deviation.

**Table 4.** Correlations of Hand-washing Practice with COVID-19-related Concerns and Depression (N = 1,350)

Variable	COVID-19-related concerns	Depression
	r (*p*)	
Hand-washing practice	.10 (.037)	–.11 (< .001)

COVID-19 = coronavirus disease 2019.

**Table 5.** Factors Affecting Hand-washing Practice in Older Adults (N = 1,350)

Variables		B	SE	t	*p*	β	Adj R^2^	F(*p*)
Sex^†^	Men	–1.02	0.16	–6.23	< .001	–.18	.07	13.55 (< .001)
Age		–0.03	0.01	–2.63	.001	–.07		
	None	–1.73	0.37	–4.67	< .001	–.20		
Education^‡^	Elementary·middle or high school	–1.04	0.28	–3.72	.001	–.15		
Perceived health status^§^	Poor	–0.87	0.21	–4.10	< .001	–.13		
COVID-19-related concerns		0.05	0.02	2.84	.005	.08		
Depression		–0.08	0.03	–2.66	.001	–.07		

SE = standard error; Adj = adjusted; COVID-19 = coronavirus disease 2019.

^†^Reference = Women (0); ^‡^Reference = ≥ College (0); ^§^Reference = Healthy (0).

## Data Availability

Please contact the corresponding author for data availability.
